# Interconnected roles of astrocytes and the blood–brain barrier in Parkinson’s disease: pathological evidence, mechanistic insights, and knowledge gaps

**DOI:** 10.3389/fnagi.2026.1765195

**Published:** 2026-03-05

**Authors:** Anna C. Stoll, Ashley S. Harms

**Affiliations:** Killion Center for Neurodegeneration and Experimental Therapeutics, University of Alabama at Birmingham, Birmingham, AL, United States

**Keywords:** astrocytes, blood-brain barrier, inflammation, neurodegeneration, Parkinson’s disease, vasculature

## Abstract

Parkinson’s disease (PD) has long been defined by α-synuclein aggregation and dopaminergic neurodegeneration, yet growing evidence indicates that non-neuronal contributors, specifically astrocytes and the blood–brain barrier (BBB), may play key roles in disease progression. Human neuropathological studies reveal BBB disruption and astrocytic abnormalities, including plasma protein extravasation, tight junction alterations, and microvascular degeneration in the substantia nigra and striatum, while neuroimaging and fluid biomarkers such as elevated QAlb, plasma GFAP, and CSF S100B further support *in vivo* vascular compromise and astrocytic reactivity. Complementary postmortem analyses highlight region- and stage-specific changes in both astrocytes and the vasculature. Mechanistic evidence from animal and cell-based models, including α-synuclein preformed fibrils, transgenic strains, and toxin-induced paradigms, demonstrates that BBB breakdown can precede or parallel dopaminergic loss, accompanied by disrupted astrocyte morphology, impaired end-feet polarization, and altered inflammatory and angiogenic signaling. Experimental manipulation of astrocytic pathways in these systems can either exacerbate or mitigate BBB dysfunction, underscoring a bidirectional astrocyte–vascular axis in PD pathology. Together, these findings position BBB and astrocytic dysfunction as intertwined processes that may amplify neurodegeneration, while underscoring critical gaps, including limited longitudinal human data, uncertain temporal ordering, and the need for integrative multimodal approaches, that must be addressed to determine whether astrocyte, BBB dysfunction drives disease progression or represents a secondary response, and whether it can be targeted for therapeutic intervention.

## Introduction

**FIGURE 1:**
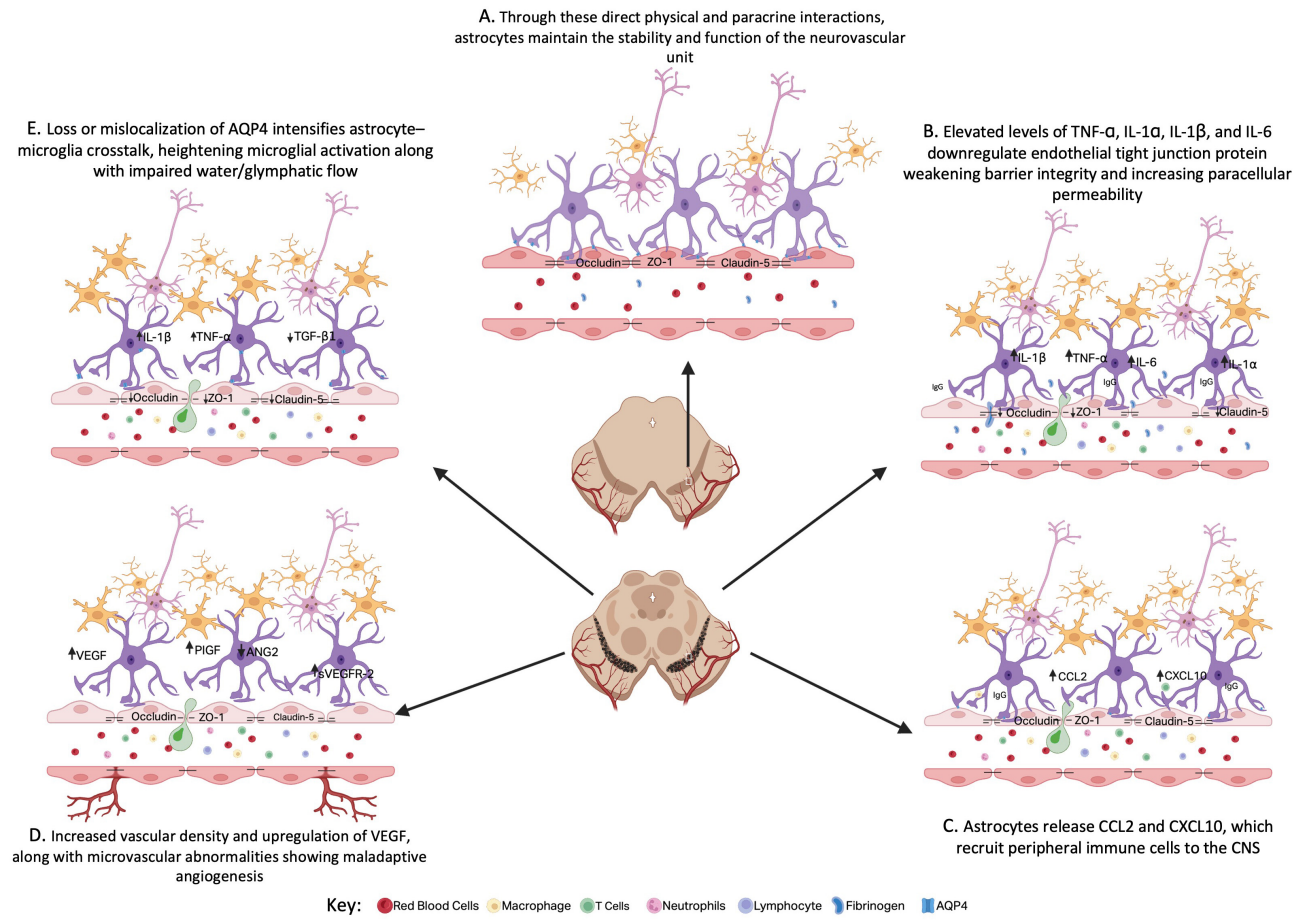
Changes in the NVU in the SNpc during disease progression. **(A)** Overview of the neurovascular unit (NVU) in health, highlighting astrocyte-blood-brain barrier interactions through both physical contacts and paracrine signaling mechanisms. **(B)** Elevated pro-inflammatory cytokines (TNF-α, IL-1α, IL-1β, IL-6) suppress endothelial tight-junction protein expression, weakening BBB integrity and increasing paracellular permeability. **(C)** At the same time, astrocyte-derived chemokines such as CCL2 and CXCL10 facilitate the recruitment of peripheral immune cells into the CNS. **(D)** Early increases in vascular density, VEGF upregulation, and microvascular abnormalities suggest maladaptive angiogenesis. **(E)** Further, disruption of astrocyte function also leads to loss or mislocalization of AQP4, which amplifies astrocyte-microglia crosstalk, heightens microglial activation, and impairs glymphatic water transport. Together, these structural and paracrine interactions highlight the central role of astrocytes in coordinating and preserving neurovascular unit stability under both physiological and pathological conditions. Figure made with UAB Liscenced BioRender.

The blood-brain barrier (BBB) is a highly selective, dynamic interface that separates the circulating blood from the brain’s extracellular environment, maintaining the delicate homeostasis essential for proper neuronal function. Composed of endothelial cells, astrocytic end-feet, pericytes, and the surrounding basement membrane, the BBB tightly regulates the transport of ions, nutrients, and signaling molecules while preventing the entry of potentially harmful substances and immune cells from the blood. Importantly, astrocytes play a central role in the BBB’s structure and regulation: their perivascular endfeet wrap around nearly the entire capillary surface, are enriched in key channels such as aquaporin-4 (AQP4) and Kir4.1 for water and ion homeostasis, and secrete signaling molecules (e.g., angiopoietin-1, sonic hedgehog, glial-derived neurotrophic factor) that promote tight junction expression between endothelial cells. Through these direct physical and paracrine interactions, astrocytes maintain the stability of the neurovascular unit and help suppress inappropriate immune cell infiltration (Figure 1A). This precise control is crucial for protecting the central nervous system (CNS) from fluctuations in the peripheral environment. However, given their participation in barrier function, when the BBB becomes compromised, changes in astrocyte functions and states may contribute to or exacerbate various neurological diseases. In neurodegenerative disorders such as Parkinson’s disease (PD), BBB changes has been increasingly recognized as a contributing factor to neuroinflammation, neuronal vulnerability, and disease progression, underscoring the importance of vascular-glial integrity (including astrocyte-endothelial interactions) in maintaining brain health.

Parkinson’s disease is a progressive neurodegenerative disorder primarily characterized by the loss of dopaminergic neurons in the substantia nigra pars compacta (SNpc), leading to the hallmark motor symptoms of tremor, rigidity, bradykinesia, and postural instability. Beyond these motor deficits, PD also encompasses a wide range of non-motor symptoms—including sleep disturbances, constipation, mood disorders, and cognitive decline—that often precede clinical diagnosis by years or even decades. At the pathological level, PD is defined by the presence of intracellular aggregates of misfolded α-synuclein (α-syn), known as Lewy bodies and Lewy neurites, which contribute to neuronal dysfunction and death. While the precise cause of PD remains elusive, mounting evidence suggests that neuroinflammatory processes, oxidative stress, and BBB leakiness play critical roles in disease onset and progression, linking vascular and immune mechanisms to neurodegeneration.

The contribution of vascular changes and BBB alterations to neurodegeneration has long been recognized in neurological disorders, and emerging evidence suggests a similar role in PD (Bower et al., 2003; Stern, 1991; Tsai et al., 2002). Observational studies indicate that regions of dopaminergic neuron loss often coincide with vascular abnormalities and astrocytic reactivity, supporting a potential mechanistic link between glial dysfunction and barrier compromise (Rite et al., 2007; Tomás-Camardiel et al., 2004). While these human studies provide critical correlational evidence, they cannot establish causality, highlighting the need for experimental models to dissect how astrocytic changes drive BBB dysfunction and influence neurodegeneration.

Given the emerging recognition of vascular contributions to neurodegeneration, growing attention has turned toward understanding how BBB integrity is altered in PD. Increasing evidence from human studies now indicates that BBB disruption may not merely be a secondary consequence of neuronal loss but an active component of disease pathology. Moreover, accumulating data suggest that functional astrocytic changes, through impaired endfeet signaling, loss of trophic support, and altered inflammatory responses, may play a pivotal role in compromising BBB stability and amplifying neurovascular inflammation. Importantly, the human patient data are observational, providing correlations but not definitive causal links between astrocyte dysfunction and BBB alterations. To address these uncertainties, this review will focus on two key questions: *How do astrocytic alterations in PD contribute to BBB disruption and vascular remodeling? What are the mechanistic links between glial dysfunction, neuroinflammation, and neuronal loss?* Exploring these questions provides crucial insight into how BBB changes, together with astrocyte-mediated alterations, contribute to inflammation, neurotoxicity, and the progression of PD.

## Blood brain barrier changes in patients with PD

### Evidence in neuroimaging and fluid biomarker analysis

Multiple human studies indicate that the BBB is compromised in PD, particularly in regions of greatest neurodegeneration. Using dynamic contrast–enhanced MRI (DCE-MRI), Al-Bachari et al. (2020) demonstrated significantly elevated K*^trans^*, a parameter reflecting BBB leakiness through contrast agent leakage, in the SNpc, white matter, and posterior cortical regions, in PD patients compared to healthy controls. Interestingly, the leakage pattern differed from that seen in individuals with cerebrovascular disease, suggesting disease specificity. PET imaging with (^11^C)-verapamil, a probe for P-glycoprotein (P-gp) efflux function, has revealed altered BBB transporter activity in some PD cohorts (Bartels et al., 2008; Kortekaas et al., 2005), although results vary depending on the disease stage and brain region, supporting the idea that BBB dynamics change as the disease progresses.

Fluid biomarker analysis further supports the idea of BBB leakiness in PD. The CSF/serum albumin quotient (QAlb), a commonly used clinical marker of BBB and blood–CSF barrier integrity, is elevated in advanced PD, suggesting increased BBB permeability (Pisani et al., 2012). However, findings in early-stage PD are more variable (Haussermann et al., 2001; Pisani et al., 2012). Consistent with this, Llorens et al. (2015) reported increased QAlb in dementia-associated synucleinopathies, including PD with dementia (PDD) and dementia with Lewy bodies (DLB), suggesting that barrier changes may intensify with disease progression and cognitive decline.

In addition to an increased QAlb, Janelidze et al. (2015) identified increased levels of angiogenesis- and endothelial-related proteins, VEGF, PlGF, and sVEGFR-2, and lower levels of Ang2, in the CSF of PD patients, correlating with white matter lesion burden on MRI. These markers were associated with gait difficulties and orthostatic hypotension. Similarly, in PDD, altered CSF vascular protein profiles have been reported, consistent with maladaptive vascular remodeling and BBB disruption (Llorens et al., 2015).

Together, neuroimaging and fluid biomarker studies provide compelling evidence that BBB integrity is altered in PD, with changes that correlate with disease progression. Imaging approaches such as DCE-MRI and PET not only demonstrate region-specific alterations but also suggest that these alterations differ from those observed in other neurological conditions, pointing toward possible disease-specific mechanisms. Complementary fluid biomarker studies further support this view, showing increased QAlb and dysregulated vascular protein profiles in PD and related synucleinopathies, particularly in advanced stages and in association with cognitive decline or vascular comorbidities. Yet, it is still unclear whether BBB fluctuations lead to neuronal injury or are a consequence of ongoing neurodegeneration. Furthermore, the mechanisms underlying regional and cellular susceptibility are not understood. While patient data provide important evidence of barrier modifications and their clinical associations, they remain limited in revealing the precise cellular and molecular mechanisms involved. Postmortem analyses complement these methods by offering direct insight into structural and molecular alterations at the NVU in PD. Despite this progress, substantial gaps in knowledge remain: longitudinal, multimodal imaging-biomarker studies are urgently needed to determine the temporal sequence of BBB breakdown relative to α-syn accumulation, neuroinflammation, and cognitive decline. In parallel, studies integrating patient-derived cellular models with *in vivo* imaging are required to link macroscopic BBB alterations to cell-type-specific mechanisms. Such approaches will be critical for establishing whether BBB changes are a driver, amplifier, or downstream consequence of PD progression.

### Evidence of BBB breakdown in postmortem tissue analysis

While neuroimaging and biomarker studies point to BBB dysfunction in PD, postmortem tissue analysis offers the most direct confirmation of widespread vascular breakdown. Immunohistochemical analyses of postmortem PD brains support the neuroimaging observations and have demonstrated the presence of extravascular blood proteins, such as IgG and fibrinogen (Gray and Woulfe, 2015; Pienaar et al., 2015; Wang et al., 2024), along with infiltration of CD8^+^ and CD4^+^ T cells in the SNpc consistent with plasma protein leakage into the parenchyma (Brochard et al., 2009; Gray and Woulfe, 2015; Lau et al., 2024). These vascular abnormalities are frequently accompanied by microvascular degeneration, perivascular plasma protein deposition (Paul and Elabi, 2022), and thickening of the basement membrane in the cingulate cortex (Farkas et al., 2000; Guan et al., 2013). Further evidence of IgG accumulation in the SN and elevated expression of IgG-binding receptors on activated microglia (Orr et al., 2005) reinforces the conclusion that BBB integrity is compromised. While BBB disruption may explain the Tcell, IgG, and fibrinogen deposition in the brain, recent literature indicates that T and B cells can reside in brain parenchyma and meninges even under non-pathological conditions. Therefore, it is possible that some immune cells are already present in the CNS or may infiltrate in an antigen-specific manner, rather than arriving solely due to barrier breakdown (Harms et al., 2021).

Complementing these findings of vascular leakage and immune infiltration, additional postmortem studies point to more subtle but functionally significant alterations in neurovascular architecture. Issidorides (1971) reported that melanin-containing neurons in the SNpc normally maintain close contact with adjacent capillaries, a structural relationship that is markedly reduced in PD. Beyond this neuronal–vascular uncoupling, more recent work has shown disorganization of critical tight-junction proteins—including claudin-5, occludin, and ZO-1—in basal ganglia microvessels (Kuan et al., 2016; Pienaar et al., 2015), further supporting impaired barrier function. Interestingly, Pienaar et al. (2015) also found that PD patients treated with subthalamic nucleus deep brain stimulation exhibited comparatively preserved microvascular integrity, suggesting that neuromodulation may influence cerebrovascular stability.

Beyond the structural BBB disruptions described above, PD brains also exhibit evidence of active vascular remodeling and maladaptive angiogenesis. Postmortem analyses reveal increased vascular density and upregulation of VEGF in the SN, frequently localized to regions of pronounced neuronal loss (Faucheux et al., 1999; Wada et al., 2006). Additional microvascular abnormalities include elevated numbers of string vessels (Yang et al., 2015) and capillaries that are fewer, shorter, dilated, and less branched (Guan et al., 2013). Although this angiogenic response is thought to reflect a stress-induced attempt to restore perfusion, the resulting neovessels often exhibit immature morphology and incomplete tight junctions, thereby contributing to further barrier instability (Desai Bradaric et al., 2012). Together, these data suggest that while angiogenesis in PD may begin as a compensatory process, it progressively becomes maladaptive, yielding structurally compromised vessels that exacerbate BBB leakiness, inflammation, and neuronal vulnerability.

Overall, these postmortem findings demonstrate that vascular pathology in PD extends beyond simple barrier leakiness to encompass immune infiltration, structural remodeling, and aberrant angiogenesis. However, it is important to acknowledge that the manner of death and associated systemic factors (e.g., hypoxia, prolonged agonal state, sepsis) can themselves alter vascular morphology and tight junction integrity in the BBB, a confounding variable in postmortem neuropathology that must be considered when interpreting structural changes. Controlled postmortem studies that carefully match PD cases to controls on key variables including age, post-mortem interval, and clinical cause of death are essential to isolate disease-specific vascular signatures from terminal systemic effects (Dik et al., 2025; Gray and Woulfe, 2015).

These cellular and molecular signatures align with *in vivo* observations of progressive BBB dysfunction, but uniquely highlight how newly formed or remodeled vessels may lack the structural integrity necessary to support neuronal health, but also highlight how confounding factors related to mode of death can impact BBB structure and should be accounted for in study designs. This raises a critical question: which cellular players drive these vascular abnormalities? Given their extensive perivascular coverage and central role in BBB regulation, astrocytes are strong candidates. Examining astrocytic changes in postmortem PD tissue therefore provides an essential link between the *in vivo* evidence of BBB disruption and the cellular mechanisms that maintain, or destabilize, vascular integrity in disease.

## Astrocytes changes seen in patients with PD

Astrocytes undergo profound morphological and biochemical changes in PD, positioning them as central contributors to neuronal vulnerability and disease progression. In response to pathological stress, astrocytes adopt a reactive phenotype characterized by hypertrophy of cell bodies, thickening and retraction of processes, and reorganization of glial fibrillary acidic protein (GFAP) -rich cytoskeletal networks (Booth et al., 2017; Dai et al., 2023; Sofroniew and Vinters, 2010; Wang and Ye, 2021). These structural alterations coincide with transcriptional and proteomic shifts that enhance inflammatory mediator release, alter neurotransmitter uptake and ion buffering, disrupt trophic support, and modify astrocyte–vascular interactions (Dai et al., 2023; Dozio and Sanchez, 2018; Siciliano et al., 2025). Clinical studies provide indirect evidence of this process through fluid biomarkers: elevated serum and CSF levels of S100β, an astrocytic protein that can leak into the bloodstream following BBB disruption and CSF during disease, have been reported in PD cohorts and correlate with disease severity, suggesting both astrocytic reactivity and vascular compromise (Carvalho et al., 2015; Papuć and Rejdak, 2020; Sathe et al., 2012). Similarly, increases in circulating GFAP support the occurrence of astrogliosis during PD progression (Chen et al., 2024; Su et al., 2012; Tang et al., 2023) possibly allowing for GFAP and S100β to be used as biomarkers in the plasma and CSF in the future (Angelopoulou et al., 2021; Lin et al., 2023; Yamashita et al., 2023).

Advances in molecular imaging have further illuminated the dynamic trajectory of astrocytic involvement across disease stages. Imidazoline-2 binding sites (I2BS), selectively expressed in reactive astrocytes, can be visualized with ^11^C-BU99008 positron emission tomography (PET) (Parker et al., 2014; Tyacke et al., 2018). Using this approach, Liu et al. (2022) demonstrated increased astrocytic activity in the cortex and brainstem during early PD, whereas patients with moderate to advanced disease exhibited reduced cortical and subcortical binding, reflecting a decline in astrocytic function as degeneration progresses (Wilson et al., 2019). Collectively, these findings portray astrocytes as dynamic players in PD pathology: initially adopting a reactive phenotype that may serve protective or compensatory purposes but ultimately undergoing functional decline that may exacerbate neuronal vulnerability and disease progression.

Histopathological analyses indicate that astrocytic alterations in PD are stage-dependent, positioning astrocytes as active contributors to disease initiation and progression. Early postmortem work in the SNpc revealed reactive astrocytes with GFAP and S100β upregulation, reflecting localized gliosis (Carvalho et al., 2015; Damier et al., 1993; Knott et al., 1999; Sathe et al., 2012). Normally, astrocytic processes ensheath neurons, providing metabolic and structural support; however, during neuronal degeneration, these processes retract, allowing activated microglia to occupy the perineuronal space—a shift from a supportive to a permissive environment for neuronal loss (Bancroft et al., 2022; Knott et al., 1999). Interestingly, GFAP expression inversely correlates with α-syn accumulation in the SNpc, suggesting that astrocytic reactivity may diminish as pathogenic protein burden increases (Tong et al., 2015).

Astrocytic involvement in PD extends beyond the CNS. Peripheral studies report reduced AQP4 and tyrosine hydroxylase (TH) mRNA levels, alongside elevated proline-rich basic protein (PBP), pointing to systemic dysregulation of astrocytic functions, including water transport and neurotransmitter metabolism (Thamizh Thenral and Vanisree, 2012). Longitudinal data from the Parkinson’s Progression Markers Initiative (PPMI) cohort further demonstrate that AQP4 genetic variation influences glymphatic efficiency, with the rs162009 variant emerging as a prognostic marker for cognitive decline in PD (Fang et al., 2022).

Further genetic evidence also underscores astrocytic vulnerability in PD. Many PD-associated genes, including ATP13A2, GBA1, LRRK2, PARK7/DJ-1, PINK1, and Parkin, are highly expressed in astrocytes (Booth et al., 2017), suggesting that astrocytes may be direct targets of molecular stressors that drive disease pathology. In addition, the SNP *rs9722* in the S100B gene is associated with increased serum S100B levels and earlier-onset PD, further linking astrocytic signaling dysregulation to disease susceptibility (Fardell et al., 2018; Hohoff et al., 2010). Given their essential roles in glutamate clearance, extracellular K^+^ buffering, BBB maintenance, antioxidant release, gliotransmission, and synaptic physiology (Chung et al., 2015; Manu et al., 2023; Mishra et al., 2017; Papouin et al., 2017; Valles et al., 2023), the convergence of genetic susceptibility, functional impairment, and regional neuropathology strongly implicates astrocytes as key determinants of α-syn–related dysfunction in PD.

Together with evidence of BBB disruption in PD, these findings highlight astrocytes as central mediators linking vascular dysfunction to neuronal vulnerability. Their strategic positioning at the NVU allows them to directly influence barrier stability and vascular remodeling. Postmortem analyses consistently reveal reduced expression of endothelial tight junction proteins, vascular leakage, and perivascular accumulation of plasma proteins in the SN and other affected regions, while transcriptomic and histopathological findings highlight astrocytic hypertrophy, inflammatory activation, and altered expression of water and ion channels. Yet, despite their frequent coexistence, the causal and temporal relationships between these vascular and glial alterations remain difficult to disentangle in human tissue. It is therefore critical to integrate findings from experimental models that permit mechanistic interrogation. The convergence of astrocytic reactivity, neuroinflammation, and endothelial compromise observed in animal and cellular systems offers an avenue to bridge these gaps—allowing us to explore how astrocyte-driven signaling, metabolic support failure, and structural remodeling of the neurovascular unit collectively shape BBB alterations in PD.

The postmortem and imaging studies described above underscore the central role of astrocytes in PD pathology, highlighting their dynamic reactivity, functional decline, and close association with the neurovascular unit. Given their intimate contact with endothelial cells through perivascular endfeet and their key roles in water and ion homeostasis, neurotransmitter clearance, and inflammatory signaling, astrocytic dysfunction is well-positioned to directly influence BBB integrity. Indeed, evidence of vascular leakage, tight junction disruption, and maladaptive angiogenesis in PD often coincides with regions of astrocytic hypertrophy and reactive gliosis, suggesting a mechanistic link between glial pathology and barrier compromise. While human tissue provides critical observational data, experimental models, ranging from cellular systems to animal models, are essential for probing causality and dissecting the molecular and cellular interactions by which astrocytes may modulate BBB function in disease.

## How can these changes in astrocytes lead to BBB breakdown?

While the precise role of astrocytes in maintaining BBB integrity remains an area of active debate—and may vary across disease contexts, accumulating evidence indicates that intact astrocytic endfeet and astrocyte-specific signaling pathways are essential for preserving BBB stability in the adult brain (Díaz-Castro et al., 2023). This section therefore focuses on astrocyte-specific mechanisms altered in PD and examines how deviations from normal astrocytic support functions, rather than a simple loss or gain of astrocyte involvement, may drive regionally selective BBB dysfunction as disease progresses.

### Astrocyte transformation to reactive and inflammatory state: secretion of cytokines, chemokines, and MMPs that disrupt endothelial tight junctions

Astrocytes adopt reactive and inflammatory states characterized by hypertrophy, GFAP upregulation, and enhanced secretion of soluble mediators that profoundly affect vascular integrity. In PD models and patient-derived systems, this transformation is thought to be driven by α-syn pathology accumulation, oxidative stress, and AQP4 dysregulation, each converging on proinflammatory signaling cascades. Elevated levels of TNF-α, IL-1α, IL-1β, and IL-6 are consistently reported, and these cytokines directly downregulate endothelial tight junction proteins—including claudin-5, occludin, and ZO-1—thereby weakening barrier integrity and increasing paracellular permeability (Figure 1B; de Rus Jacquet et al., 2023; Lan et al., 2021; Lau et al., 2023). In human LRRK2 G2019S astrocytes, IL-6 secretion correlates with both neuronal toxicity and failure to support BBB formation *in vitro*, highlighting the causal impact of astrocyte-derived inflammatory mediators on barrier function (de Rus Jacquet et al., 2023).

Reactive astrocytes also release chemokines such as CCL2 and CXCL10, which recruit peripheral immune cells to the CNS, amplifying neuroinflammation and further destabilizing the BBB (Lau et al., 2024). These effects are reinforced by AQP4-dependent signaling: loss or mislocalization of AQP4 intensifies astrocyte–microglia crosstalk, heightening microglial activation and promoting release of IL-1β and TNF-α (Sun et al., 2016; Xue et al., 2019). This proinflammatory loop reduces astrocytic TGF-β1 production, removing an important anti-inflammatory restraint and can exacerbate vascular and neuronal injury (Figure 1C).

Beyond cytokines and chemokines, reactive astrocytes secrete vascular- and protease-related effectors that further compromise the BBB. In α-syn models, oligomeric α-syn stimulates astrocyte-derived vascular endothelial growth factor A (VEGFA) and nitric oxide (NO) production, which increase endothelial permeability and promote tight junction disassembly; blockade of VEGFA signaling rescues barrier integrity (Lan et al., 2021). Additionally, matrix metalloproteinases (MMPs) add a proteolytic component to this injury. MMP-2, MMP-3, and MMP-9 are upregulated in multiple PD models—particularly MMP-3 in Thy1-αSyn mice, where its induction coincides with reduced occludin expression and evidence of vascular leakage (Lau et al., 2023). Notably, however, Lau et al. (2023) report several counterintuitive features: despite measurable changes in occludin and other tight junction proteins, widespread extravasation of large serum proteins such as IgG is limited under basal conditions, and alterations in tight junction components are heterogeneous across vascular segments and brain regions. These findings suggest that tight junction protein loss alone is not sufficient to drive overt BBB breakdown in α-synucleinopathy, and instead point to a more nuanced, potentially threshold-dependent barrier dysfunction that may require additional inflammatory or metabolic stressors to manifest as frank leakage. Consistent with this view, MMP-mediated degradation of basal lamina components such as laminin and collagen IV may prime the neurovascular unit for permeability changes without immediately producing classical markers of barrier failure (Lakhan et al., 2013; Sorrentino et al., 2020).

Consistent alterations are evident in PD patient tissue: reduced claudin-5 and occludin expression in SN microvessels, together with increased fibrinogen and IgG deposition in the parenchyma, indicate BBB leakage (Gray and Woulfe, 2015; Pisani et al., 2012). Elevated MMP-9 levels in PD serum and cerebrospinal fluid correlate with disease severity, supporting a role for glial-derived proteolysis in human BBB disruption (Chao et al., 2014).

Together, these findings demonstrate that reactive astrocytes are not passive responders but active drivers of BBB pathology. Through a secretory profile combining cytokine- and chemokine-mediated endothelial changes, VEGFA/NO-dependent permeability signaling, and MMP-driven basal lamina degradation, astrocytes create a permissive environment for blood-derived proteins and immune cells to infiltrate the brain parenchyma. This dual inflammatory–proteolytic mechanism establishes a chronic neurovascular inflammatory milieu that can contribute to the progression of PD pathology.

### Astrocyte-mediated dysregulation of vascular maintenance and remodeling in PD

Astrocytes play a central role in maintaining vascular structure and health by releasing trophic and regulatory molecules—including TGF-β1, GDNF, angiopoietin-1, sonic hedgehog (Shh), VEGF, and MMPs, that support endothelial survival, tight junction expression, extracellular matrix remodeling, and angiogenesis (Michinaga and Koyama, 2019; Spampinato et al., 2019; Zhang et al., 2023). These signals collectively sustain capillary networks, preserve BBB stability, and promote vascular homeostasis. In PD, astrocytes frequently lose this homeostatic phenotype under conditions of α-syn stress, inflammation, or aging. The resulting imbalance, characterized by reduced trophic support (e.g., TGF-β1, GDNF, angiopoietin-1, Shh) and maladaptive upregulation of VEGF and related angiogenic factors, leads to both BBB fragility and pathological vascular remodeling. This shift drives increased vessel permeability, abnormal microvessel formation, and eventual capillary rarefaction, compounding neuronal vulnerability (Bezard et al., 2003; Lan et al., 2021; Lau et al., 2023; Sha et al., 2024).

Experimental and patient-based evidence supports this dual mechanism. In α-syn A53T transgenic mice and AAV-mediated α-syn overexpression models, elevated astrocytic VEGF expression correlated with loss of endothelial tight junction proteins, perivascular α-syn accumulation, and widespread microvascular pathology (Lan et al., 2021; Lau et al., 2024). Human BBB-on-chip studies demonstrate that astrocytes carrying LRRK2 mutations fail to maintain endothelial networks (de Rus Jacquet et al., 2023), while postmortem analyses of PD brains reveal vessel degeneration in the SN and cortex, reduced microvessel density, and progression from early BBB disruption and compensatory angiogenesis to eventual vascular rarefaction (Cabezas et al., 2014; Guan et al., 2013; Paul and Elabi, 2022). Similar findings have been observed in MPTP-treated nonhuman primates, where increased VEGF expression corresponded with abnormal vascularization (Barcia et al., 2005), and CSF biomarkers in PD patients reveal elevated VEGF and PlGF levels, indicative of ongoing but maladaptive angiogenic signaling (Figure 1D; Janelidze et al., 2015).

Together, these studies indicate that astrocytes contribute to PD-related vascular pathology through both loss of homeostatic trophic and maintenance signals and dysregulated angiogenic remodeling. The combination of reduced supportive cues and overactive VEGF-mediated signaling produces leaky, structurally abnormal microvessels that exacerbate BBB breakdown, microvascular degeneration, and neuronal vulnerability, highlighting astrocytes as critical modulators of neurovascular health and promising therapeutic targets in PD.

### Loss and mis localization of AQP4 in astrocyte end feet may lead to impaired water/glymphatic flow and weakened BBB structure

Loss and mislocalization of AQP4 in astrocyte end-feet may lead to impaired water and glymphatic flow and weakened BBB structure. AQP4 is highly concentrated in perivascular astrocytic end-feet, where it regulates water transport, supports glymphatic clearance, and maintains astrocyte–endothelial coupling. Disruption of this localization, either through reduced perivascular coverage, abnormal distribution, or phosphorylation changes, has been observed in both PD models and patient-relevant studies (Figure 1E; Booth et al., 2017; Braun et al., 2024; Cui et al., 2021; Lapshina and Ekimova, 2024).

In toxin-induced parkinsonian models, dysregulation of AQP4 has been shown to exacerbate disease progression and neuroinflammation. In MPTP-treated mice, AQP4 deficiency worsened dopaminergic neurodegeneration, enhanced microglial activation, and reduced astrocytic production of the anti-inflammatory cytokine TGF-β1 (Fan et al., 2008; Subburaman, 2010; Xue et al., 2019). Moreover, loss of AQP4 impaired astrocyte–microglia communication, resulting in heightened gliosis and increased IL-1β and TNF-α expression during chronic MPTP exposure (Sun et al., 2016). Conversely, in the 6-OHDA model, AQP4 expression was increased and associated with astrocytic hypertrophy, perivascular edema, and tight junction loss (Vizuete et al., 1999; Wachter et al., 2012), suggesting that both deficiency and dysregulated upregulation of AQP4 can destabilize BBB homeostasis.

In α-syn models, reduced or mislocalized AQP4 expression has been consistently linked to impaired vascular integrity and glymphatic clearance. In Thy1-αSyn transgenic mice, AQP4 loss at astrocytic end-feet indicated compromised perivascular coupling and BBB support (Lau et al., 2023). Similarly, in α-syn preformed fibril (PFF) models, reduced or aberrantly localized AQP4 accelerated α-syn aggregation, dopaminergic neuron loss, and behavioral deficits, in part through impaired glymphatic clearance of macromolecules (Braun et al., 2024; Cui et al., 2021).

Collectively, these findings demonstrate that AQP4 plays a dual role as both a regulator of brain fluid dynamics and a structural stabilizer of the neurovascular unit. Loss or mislocalization of AQP4 compromises interstitial fluid and solute clearance, facilitating extracellular accumulation of toxic proteins such as α-syn and contributing to cerebral edema. At the same time, weakened end-foot anchoring undermines vascular basement membrane stability and disrupts tight junction protein organization, increasing BBB permeability and vascular leakage (Lan et al., 2021; Lau et al., 2023). Thus, alterations in AQP4 not only impair glymphatic flow but also erode the structural and homeostatic support astrocytes provide to the cerebrovasculature, linking astrocytic modifications to both protein aggregation and BBB changes in PD.

### Astrocytic responses to neuronal α-syn pathology and signaling with pericytes, driving perivascular inflammation and BBB disruption

Although α-syn accumulation and aggregation occur primarily within neurons, the resulting pathogenic environment exerts profound secondary effects on astrocytes, pericytes, and endothelial cells within the neurovascular unit. Increasing evidence indicates that the strain and structural conformation of α-syn, particularly oligomeric species, contributes to selective neuronal vulnerability and to the downstream non-cell-autonomous responses observed in surrounding glial and vascular cells (Bengoa-Vergniory et al., 2017; Lau et al., 2020; Van der Perren et al., 2020). Oligomeric α-syn, which is both present in PD patient tissue and found to be neurotoxic in transgenic models, elicits far more potent glial activation than monomeric or fibrillar forms. *In vitro* BBB models further show that oligomeric α-syn specifically (not monomeric or fibrillar species) disrupts BBB integrity through astrocyte-dependent mechanisms, implicating pathogenic neuronal α-syn species as key upstream initiators of vascular fluctuations (Lan et al., 2021).

Neuronal α-syn pathology drives astrocyte activation through possible release of inflammatory and oxidative stress signals and exposure to extracellular oligomeric species, triggering NF-κB–dependent transcription, cytokine production, and in some cases inflammatory senescence (Chou et al., 2021; Huang et al., 2022; Jeon et al., 2025; Sorrentino et al., 2017, 2020). Astrocytes in α-syn-rich regions exhibit AQP4 mislocalization, diminished end-foot coverage, and impaired metabolic and structural support for vessels (Lan et al., 2021; Lau et al., 2023). Importantly, reactive transformation also compromises astrocytic proteostasis and debris-handling capacity. Under pathological α-syn and inflammatory stress, astrocytes show reduced efficiency in clearing extracellular aggregates and neuronal debris, functions normally mediated through endolysosomal pathways and phagocytic-like uptake. This impaired clearance not only allows pathogenic α-syn species to persist in the extracellular space but also increases the burden of damage-associated molecular patterns (DAMPs), further amplifying microglial activation, cytokine release, and vascular inflammation (Chang et al., 2024; Kim et al., 2023). Thus, deficits in astrocytic debris clearance contribute an additional feed-forward mechanism that exacerbates perivascular stress and destabilizes the BBB (Lau et al., 2024; Sui et al., 2014).

Once activated, astrocytes secrete a broad array of inflammatory and vasoactive mediators—including VEGFA and nitric oxide (NO)—which have been directly linked to endothelial permeability increases and tight-junction disassembly. Elevated VEGFA and iNOS levels have been observed in PD patient brains, and blocking VEGFA signaling restores BBB integrity *in vitro* and in mouse models, demonstrating that astrocyte-mediated VEGFA/NO signaling is a key pathway connecting neuronal α-syn pathology to BBB dysfunction (Lan et al., 2021).

Pericytes also respond robustly to the inflammatory and oxidative signals generated in α-syn–affected regions. Exposure to α-syn–induced astrocyte cytokines or broader neuronal stress cues activates pericytes to secrete proinflammatory factors and MMPs, producing rapid reductions in endothelial barrier resistance and accelerating junctional degradation (Dohgu et al., 2019; Stevenson et al., 2022). Endothelial cells likewise activate stress and inflammatory pathways in response to the glial environment shaped by neuronal α-syn burden. Together, astrocyte-derived cytokines and VEGFA/NO, pericyte activation, AQP4-related end-foot changes, and impaired clearance of extracellular debris converge to generate a perivascular inflammatory milieu that promotes paracellular leak, immune cell infiltration, and persistent BBB instability.

Across co-culture systems, iPSC-derived neurovascular models, and α-syn overexpression or PFF exposure paradigms in animals, these neuronal α-syn–initiated glial and vascular interactions consistently produce measurable BBB compromise, including reduced tight-junction expression, heightened inflammatory signaling, and tracer leakage (Lan et al., 2021; Lau et al., 2023; Pediaditakis et al., 2021). Collectively, these findings support a model in which neuronal α-syn pathology, particularly oligomeric species, acts as the upstream driver of astrocyte activation and pericyte alterations, with these secondary cellular responses serving as critical mediators of BBB disruption in synucleinopathies (Hourfar et al., 2023)

Together, these findings outline a compelling model in which neuronal α-syn pathology, especially oligomeric species, initiates a cascade of non–cell-autonomous responses that ultimately destabilize the BBB through astrocytic, pericytic, and endothelial changes. Yet despite this converging evidence, major gaps remain. The precise neuronal signals that trigger astrocytic activation, the molecular determinants that differentiate protective from pathogenic glial responses, and the sequence by which pericytes and endothelial cells succumb to α-syn–driven inflammatory stress are still poorly defined. Equally unclear is the extent to which impaired astrocytic debris clearance, now recognized as a contributor to perivascular inflammation, represents an early, potentially reversible event or a late-stage consequence of overwhelmed glial proteostasis. Moreover, the relative contribution of α-syn strain conformation, regional neuronal subtype vulnerability, and aging-associated changes within the neurovascular unit remains largely unexplored. Future work will require integrated *in vivo*, human iPSC–derived, and spatial multiomics approaches to dissect how neuronal α-syn pathology is communicated across cell types, to pinpoint the earliest reversible steps in BBB decline, and to determine whether targeting astrocyte–pericyte–endothelial signaling can meaningfully halt or slow neurodegeneration. Clarifying these unresolved mechanisms is essential for translating the emerging neuron-to-vascular framework of PD into effective therapeutic strategies.

### Astrocytic endfeet calcium and mitochondrial dysregulation as contributors to BBB dysfunction

Disruptions in astrocytic calcium (Ca^2+^) signaling are increasingly recognized as a contributor to BBB dysfunction in PD. Post-mortem human studies show altered expression of L-type voltage-gated calcium channels and calcium-binding proteins, including calbindin and calmodulin, with increased CaV1.3 expression in cortical tissue detectable at early disease stages, indicating that calcium dysregulation is not merely secondary to neurodegeneration (Hurley et al., 2013). Patient-derived astrocytes carrying LRRK2 G2019S or GBA N370S mutations display abnormal intracellular Ca^2+^ handling, including altered endoplasmic reticulum calcium release and elevated basal calcium levels (Sonninen et al., 2020; Woodard et al., 2014). Consistent with these human data, animal models further demonstrate that astrocytes in α-syn A53T transgenic mice and LRRK2 G2019S mice show dysregulated Ca^2+^ dynamics, altered gliotransmission, and impaired neurotrophic factor release, linking calcium dysfunction in endfeet to compromised neurovascular interactions (Lee et al., 2019; Nanclares et al., 2023). Importantly, calcium dysregulation appears particularly relevant at the level of astrocytic endfeet, as aging mouse striatum shows that spontaneous Ca^2+^ events in astrocytic endfeet, which are critical for vasoregulation and coupling to endothelial cells, change dramatically with age, including alterations in both membrane-associated and mitochondrial Ca^2+^ signals and reduced expression of key calcium buffering proteins, suggesting that endfeet Ca^2+^ dysregulation may weaken NVU function and BBB support during neurodegeneration (Zarate et al., 2023). Further, converging evidence supports the idea that altered astrocytic Ca^2+^ signaling could impact BBB integrity in PD by modifying the secretion of neurotrophic factors such as glial-derived neurotrophic factor (GDNF) and by dysregulating interactions with endothelial tight junction protein networks, changes that are plausibly mediated at the endfeet level (Bancroft and Srinivasan, 2022; Pivoriûnas and Verkhratsky, 2021). This interpretation is further supported by evidence that astrocytes contribute directly to BBB formation, in part through paracrine signaling mechanisms that regulates endothelial tight junction expression and permeability (Wang et al., 2023). Accordingly, disruption of astrocyte endfeet signaling, whether through Ca^2+^ dysregulation or altered mitochondria-linked metabolic support, has the potential to compromise barrier integrity.

At a mechanistic level, mitochondrial dysfunction, tightly connected to local Ca^2+^ handling, has been implicated in astrocyte dysfunction in PD models, including impaired glutamate metabolism, altered oxidative balance, and disturbed ion homeostasis (Bantle et al., 2021). Patient iPSC-derived astrocytes carrying PD-linked mutations, including LRRK2 G2019S and GBA variants, exhibit altered oxidative metabolism, impaired mitochondrial respiration, and disrupted energy handling, consistent with compromised oxidative phosphorylation capacity (Sonninen et al., 2020). Complementing these human data, astrocyte-focused studies demonstrate that exposure to pathological α-syn and PD-relevant genetic stressors induces mitochondrial dysfunction in astrocytes, including changes in mitochondrial morphology, bioenergetic stress, and impaired calcium–mitochondria coupling (Lindström et al., 2017; Wang et al., 2021). Aberrant astrocytic calcium signaling, which is tightly linked to mitochondrial function, is increasingly recognized in PD and is proposed to exacerbate mitochondrial stress and metabolic insufficiency in astrocytes, with downstream consequences for neurovascular support (Bancroft and Srinivasan, 2022). Together, these findings underscore that endfeet calcium signaling and mitochondrial health are not only interdependent but also crucial for the dynamic support that astrocytes provide to the neurovascular unit gliovascular signaling, PD-associated mitochondrial and Ca^2+^ signaling dysfunction in astrocytes is likely to impair endfeet–endothelial interactions and weaken BBB integrity.

## Current unknowns and unresolved questions

### Causality: astrocyte dysfunction or BBB breakdown?

**FIGURE 2:**
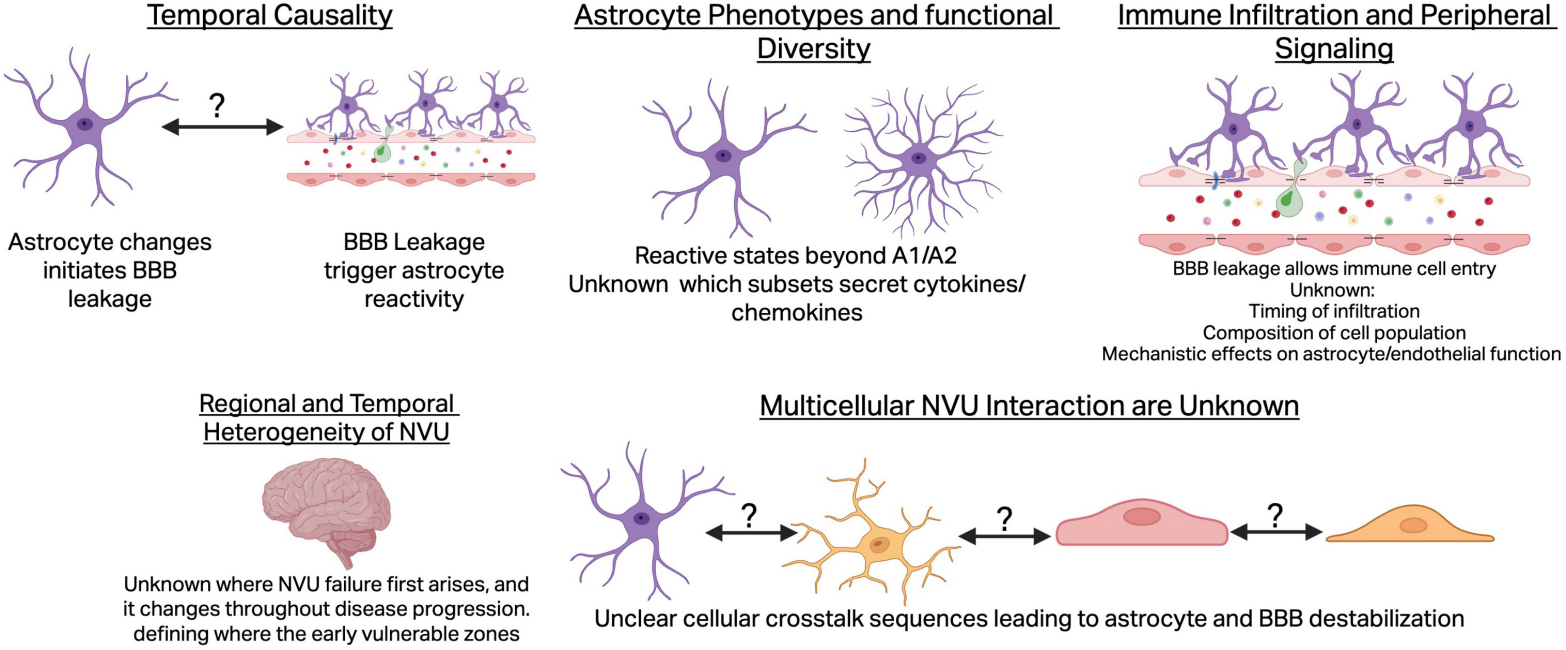
Key outstanding questions in astrocyte–blood–brain barrier (BBB) pathophysiology in Parkinson’s disease. Causality: both astrocyte reactivity and BBB leakage are well-documented in Parkinson’s disease (PD), but it remains unclear whether astrocytic dysfunction initiates barrier breakdown or arises secondarily from vascular leakage. Astrocyte phenotypes: the astrocyte subtypes responsible for BBB disruption are unknown, as single-cell studies reveal diverse reactive states beyond the A1/A2 model. Immune contributions: peripheral immune cells infiltrate the CNS after BBB leakage and may modulate astrocyte and endothelial signaling, yet their timing and mechanistic roles in PD are poorly defined. Regional and temporal heterogeneity: NVU structure varies across brain regions, yet most studies assess global permeability rather than focusing on PD-vulnerable areas like the substantia nigra. Multicellular NVU interactions: Barrier failure results from coordinated interactions among astrocytes, endothelial cells, pericytes, and microglia, but PD studies typically examine these cells in isolation. Figure made with UAB Liscenced BioRender.

Although considerable progress has been made in understanding the role of astrocytes’ dysfunction and BBB impairments in PD initiation and progression, major gaps remain in defining causality, mechanisms, and translational significance (Figure 2). Astrocytes are essential regulators of BBB function through their end-feet, which encase the vasculature and maintain endothelial tight junctions and ion homeostasis (Díaz-Castro et al., 2023). Yet it is still unclear whether functional astrocyte changes represent a cause or a consequence of BBB decline in PD. Human imaging and post-mortem studies consistently reveal both astrocyte reactivity and barrier leakage, but cross-sectional designs prevent determination of directionality. Astrocytes may lose their ability to sustain tight junctions and basement membrane integrity, thereby initiating BBB failure, or alternatively, BBB leakage of plasma proteins and inflammatory mediators may induce secondary astrocytic reactivity (Lau et al., 2024). Establishing temporal causality in humans is essential to identify whether astrocytes are early therapeutic targets or downstream responders to vascular injury.

### Astrocyte phenotypes and functional diversity

Which astrocyte phenotypes or reactive states drive barrier loss remains unresolved. The traditional binary classification into A1 (neurotoxic) and A2 (neuroprotective) astrocytes oversimplifies the transcriptional and functional diversity now evident from single-cell studies (Brash-Arias et al., 2024), which show a spectrum of astrocyte responsiveness. Reactive astrocytes have heterogeneous responses including secretion of pro-inflammatory cytokines and matrix metalloproteinases that degrade basement membrane components and disrupt endothelial tight junctions, along with expression of angiogenic, metabolic, and trophic programs that support vascular remodeling and BBB repair (Alvarez et al., 2013; Anderson et al., 2014; Brash-Arias et al., 2024; Michinaga and Koyama, 2019; Spampinato et al., 2019). Human iPSC-derived astrocytes harboring the LRRK2-G2019S mutation demonstrate impaired support of endothelial tube formation and pro-inflammatory signaling *in vitro* (de Rus Jacquet et al., 2023), implicating specific astrocyte phenotypes in NVU destabilization. However, a comprehensive mapping of astrocyte subtypes in PD brain tissue, and their spatial or temporal correlation with BBB failure, remains lacking.

### Immune contributions to BBB dysfunction

While the NVU has rarely been examined as an integrated multicellular network, the role of peripheral immune cells in BBB and astrocytic alterations remains limitedly understood. BBB leakage allows immune cells to infiltrate perivascular niches, where they may release cytokines that modulate astrocyte and endothelial function. Although immune activation and T-cell infiltration have been reported in PD (Brochard et al., 2009; Williams et al., 2021), the timing, composition, and mechanistic consequences of these infiltrates on NVU stability remain poorly defined.

### Regional and temporal heterogeneity

A further challenge lies in regional and temporal heterogeneity. The NVU is not uniform across the brain; endothelial specialization, pericyte density, and astrocyte morphology vary regionally. PD pathology itself follows a selective pattern, affecting the SN, olfactory bulb, and brainstem early, yet most human studies measure global BBB permeability rather than region- or stage-specific changes (Lau et al., 2024). Without precise mapping of where and when astrocyte–vascular changes first arise, it is difficult to identify early vulnerable zones or critical disease windows for intervention.

### Multicellular interactions within the NVU

Interactions among astrocytes, pericytes, microglia, and endothelial subtypes within the NVU are poorly characterized in PD. Barrier integrity emerges from tightly coordinated crosstalk among these cells, yet most studies isolate different cellular dysfunctions rather than investigating the multicellular network. Evidence from other neurodegenerative and vascular disorders shows that pericyte loss destabilizes the BBB, microglial activation induces astrocyte reactivity, and endothelial heterogeneity influences barrier resilience (Cabezas et al., 2014). The lack of similar data in PD limits understanding of the full cellular chain driving vascular pathology.

### Translational gaps and human relevance

A significant translational gap remains between preclinical discoveries and human pathology. Although human studies demonstrate astrocytic and BBB changes, most mechanistic insights from rodent and iPSC-based models have yet to achieve clinical validation or therapeutic translation. Emerging human-relevant systems—such as patient-derived BBB-on-chip and organoid platforms—are beginning to bridge this divide, revealing that PD astrocytes can impair barrier integrity and neurovascular signaling (de Rus Jacquet et al., 2023). Nevertheless, robust *in vivo* biomarkers and mechanistic clinical studies are still needed to determine whether these pathways function similarly in human PD and to guide the development of targeted neurovascular interventions.

## Caveats and contrasting evidence on BBB involvement in PD

While all the aforementioned studies point to changes in BBB being seen with PD, there are a few studies that do not support a widespread or robust disruption of the BBB in Parkinson’s disease. In some clinical investigations using *in vivo* imaging, evidence for altered BBB permeability is limited: for example, ^82^Rb PET studies in PD patients did not detect significant differences in rubidium influx, a proxy for BBB permeability, compared with healthy controls, suggesting no marked increase in BBB leakage in PD with or without levodopa-induced dyskinesia (except a localized change in the thalamus) (Fujita et al., 2021) and highlighting that any barrier changes might be highly focal or below detection thresholds of certain imaging modalities. Moreover, while BBB leakage has been detected by other methods in some cohorts, *in vivo* PET evidence of *significant* permeability changes remains inconsistent in PD patients, indicating that barrier dysfunction in human PD may not be as pronounced or global as in animal models (Gray and Woulfe, 2015). These findings underscore the importance of reconciling variable outcomes across methods and disease stages and suggest that discrepancies in detecting BBB effects may stem from differences in sensitivity of imaging modalities, regional specificity of barrier alterations, or heterogeneity in PD pathology among subjects.

## Conclusion

In conclusion, while mounting evidence positions astrocyte dysfunction and BBB impairment at the heart of PD pathophysiology, critical questions remain unresolved. The precise sequence of cellular events, the molecular determinants of astrocyte and pericyte responses, the basis of regional vulnerability, and the interplay with immune signaling are still poorly understood. Resolving these gaps is essential not only to delineate the mechanisms driving NVU failure, but also to identify precise, human-relevant targets for interventions aimed at preserving vascular integrity and slowing neurodegeneration in PD. Future research should prioritize longitudinal and multimodal studies in human patients to establish temporal causality, complemented by mechanistic investigations using advanced models such as transgenic animals (Hélie-Legoupil et al., 2025) iPSC-derived NVUs (Jagadeesan et al., 2020), organoids (Elvira et al., 2025), and BBB-on-chip platforms (de Rus Jacquet et al., 2023). Integration of single-cell transcriptomics, spatial mapping, and *in vivo* imaging will be critical to define the heterogeneity of astrocyte and endothelial responses across brain regions and disease stages. Furthermore, therapeutic strategies targeting astrocyte-BBB interactions—such as modulation of AQP4 localization, tight junction stabilization, or selective regulation of reactive astrocyte phenotypes—represent promising avenues for slowing disease progression and preserving neurovascular function. By addressing these future directions, the field can move toward a more mechanistic and translational understanding of astrocyte-mediated vascular pathology in PD.

## References

[B1] Al-Bachari S. Naish J. H. Parker G. J. M. Emsley H. C. A. Parkes L. M. (2020). Blood–Brain barrier leakage is increased in Parkinson’s disease. *Front. Physiol.* 11:593026. 10.3389/fphys.2020.593026 33414722 PMC7784911

[B2] Alvarez J. I. Katayama T. Prat A. (2013). Glial influence on the blood brain barrier. *Glia* 61 1939–1958. 10.1002/glia.22575 24123158 PMC4068281

[B3] Anderson M. A. Ao Y. Sofroniew M. V. (2014). Heterogeneity of reactive astrocytes. *Neurosci. Lett.* 565 23–29. 10.1016/j.neulet.2013.12.030 24361547 PMC3984948

[B4] Angelopoulou E. Paudel Y. N. Piperi C. (2021). Emerging role of S100B protein implication in Parkinson’s disease pathogenesis. *Cell. Mol. Life Sci. CMLS* 78 1445–1453. 10.1007/s00018-020-03673-x 33052436 PMC11073186

[B5] Bancroft E. A. De La Mora M. Pandey G. Zarate S. M. Srinivasan R. (2022). Extracellular S100B inhibits A-type voltage-gated potassium currents and increases L-type voltage-gated calcium channel activity in dopaminergic neurons. *Glia* 70 2330–2347. 10.1002/glia.24254 35916350 PMC10738449

[B6] Bancroft E. A. Srinivasan R. (2022). Emerging roles for aberrant astrocytic calcium signals in Parkinson’s disease. *Front. Physiol.* 12:812212. 10.3389/fphys.2021.812212 35087422 PMC8787054

[B7] Bantle C. M. Hirst W. D. Weihofen A. Shlevkov E. (2021). Mitochondrial dysfunction in astrocytes: A role in Parkinson’s disease? *Front. Cell Dev. Biol.* 8:608026. 10.3389/fcell.2020.608026 33537300 PMC7849831

[B8] Barcia C. Bautista V. Sánchez-Bahillo Á Fernández-Villalba E. Faucheux B. Poza y Poza M. (2005). Changes in vascularization in substantia nigra pars compacta of monkeys rendered parkinsonian. *J. Neural Trans.* 112 1237–1248. 10.1007/s00702-004-0256-2 15666038

[B9] Bartels A. L. Willemsen A. T. M. Kortekaas R. de Jong B. M. de Vries R. de Klerk O. (2008). Decreased blood–brain barrier P-glycoprotein function in the progression of Parkinson’s disease, PSP and MSA. *J. Neural Trans.* 115 1001–1009. 10.1007/s00702-008-0030-y 18265929 PMC2468317

[B10] Bengoa-Vergniory N. Roberts R. F. Wade-Martins R. Alegre-Abarrategui J. (2017). Alpha-synuclein oligomers: A new hope. *Acta Neuropathol.* 134 819–838. 10.1007/s00401-017-1755-1 28803412 PMC5663814

[B11] Bezard E. Baufreton J. Owens G. Crossman A. R. Dudek H. Taupignon A. (2003). Sonic hedgehog is a neuromodulator in the adult subthalamic nucleus. *FASEB J.* 17 2337–2338. 10.1096/fj.03-0291fje 14525941

[B12] Booth H. D. E. Hirst W. D. Wade-Martins R. (2017). The role of astrocyte dysfunction in Parkinson’s disease pathogenesis. *Trends Neurosci.* 40 358–370. 10.1016/j.tins.2017.04.001 28527591 PMC5462417

[B13] Bower J. H. Maraganore D. M. Peterson B. J. McDonnell S. K. Ahlskog J. E. Rocca W. A. (2003). Head trauma preceding PD. *Neurology* 60 1610–1615. 10.1212/01.WNL.0000068008.78394.2C 12771250

[B14] Brash-Arias D. García L. I. Pérez-Estudillo C. A. Rojas-Durán F. Aranda-Abreu G. E. Herrera-Covarrubias D. (2024). The role of astrocytes and alpha-synuclein in Parkinson’s disease: A review. *NeuroScience* 5 71–86. 10.3390/neurosci5010005 39483813 PMC11523690

[B15] Braun M. Simon M. J. Jang J. Sanderson K. Swierz J. Sevao M. (2024). Aquaporin-4 mis-localization slows glymphatic clearance of α-synuclein and promotes α-synuclein pathology and aggregate propagation. *bioRxiv [Preprint]* 10.1101/2024.08.14.607971 39229234 PMC11370328

[B16] Brochard V. Combadière B. Prigent A. Laouar Y. Perrin A. Beray-Berthat V. (2009). Infiltration of CD4 + lymphocytes into the brain contributes to neurodegeneration in a mouse model of Parkinson disease. *J. Clin. Invest.* 119 182–192. 10.1172/JCI36470 19104149 PMC2613467

[B17] Cabezas R. Ãvila M. Gonzalez J. El-Bachá R. S. Báez E. García-Segura L. M. (2014). Astrocytic modulation of blood brain barrier: Perspectives on Parkinson’s disease. *Front. Cell. Neurosci.* 8:211. 10.3389/fncel.2014.00211 25136294 PMC4120694

[B18] Carvalho D. Z. Schönwald S. V. Schumacher-Schuh A. F. Braga C. W. Souza D. O. Oses J. P. (2015). Overnight S100B in Parkinson’s disease: A glimpse into sleep-related neuroinflammation. *Neurosci. Lett.* 608 57–63. 10.1016/j.neulet.2015.10.010 26453767

[B19] Chang N. P. DaPrano E. M. Lindman M. Estevez I. Chou T.-W. Evans W. R. (2024). Neuronal DAMPs exacerbate neurodegeneration via astrocytic RIPK3 signaling. *JCI Insight* 9:e177002. 10.1172/jci.insight.177002 38713518 PMC11382884

[B20] Chao Y. Wong S. C. Tan E. K. (2014). Evidence of inflammatory system involvement in Parkinson’s disease. *BioMed. Res. Intern.* 2014:308654. 10.1155/2014/308654 25050341 PMC4094726

[B21] Chen T. Dai Y. Hu C. Lin Z. Wang S. Yang J. (2024). Cellular and molecular mechanisms of the blood–brain barrier dysfunction in neurodegenerative diseases. *Fluids Barriers CNS* 21:60. 10.1186/s12987-024-00557-1 39030617 PMC11264766

[B22] Chou T.-W. Chang N. P. Krishnagiri M. Patel A. P. Lindman M. Angel J. P. (2021). Fibrillar α-synuclein induces neurotoxic astrocyte activation via RIP kinase signaling and NF-κB. *Cell Death Dis.* 12:756. 10.1038/s41419-021-04049-0 34333522 PMC8325686

[B23] Chung W.-S. Allen N. J. Eroglu C. (2015). Astrocytes control synapse formation, function, and elimination. *Cold Spring Harb. Perspect. Biol.* 7:a020370. 10.1101/cshperspect.a020370 25663667 PMC4527946

[B24] Cui H. Wang W. Zheng X. Xia D. Liu H. Qin C. (2021). Decreased AQP4 expression aggravates ɑ-Synuclein pathology in Parkinson’s disease mice, possibly via impaired glymphatic clearance. *J. Mol. Neurosci.* 71 2500–2513. 10.1007/s12031-021-01836-4 33772424

[B25] Dai D. L. Li M. Lee E. B. (2023). Human Alzheimer’s disease reactive astrocytes exhibit a loss of homeostastic gene expression. *Acta Neuropathol. Commun.* 11:127. 10.1186/s40478-023-01624-8 37533101 PMC10398957

[B26] Damier P. Hirsch E. C. Zhang P. Agid Y. Javoy-Agid F. (1993). Glutathione peroxidase, glial cells and Parkinson’s disease. *Neuroscience* 52 1–6. 10.1016/0306-4522(93)90175-F 8433802

[B27] de Rus Jacquet A. Alpaugh M. Denis H. L. Tancredi J. L. Boutin M. Decaestecker J. (2023). The contribution of inflammatory astrocytes to BBB impairments in a brain-chip model of Parkinson’s disease. *Nat. Commun.* 14:3651. 10.1038/s41467-023-39038-8 37339976 PMC10282096

[B28] Desai Bradaric B. Patel A. Schneider J. A. Carvey P. M. Hendey B. (2012). Evidence for angiogenesis in Parkinson’s disease, incidental Lewy body disease, and progressive supranuclear palsy. *J. Neural Trans.* 119 59–71. 10.1007/s00702-011-0684-8 21748523 PMC3352316

[B29] Díaz-Castro B. Robel S. Mishra A. (2023). Astrocyte endfeet in brain function and pathology: Open questions. *Ann. Rev. Neurosci.* 46 101–121. 10.1146/annurev-neuro-091922-031205 36854317

[B30] Dik D. Halliday G. M. Sytnyk V. Shepherd C. E. (2025). Region-specific variations in the cerebrovasculature underlie disease progression in Parkinson’s disease. *Brain* 149 592–605. 10.1093/brain/awaf305 41285171

[B31] Dohgu S. Takata F. Matsumoto J. Kimura I. Yamauchi A. Kataoka Y. (2019). Monomeric α-synuclein induces blood–brain barrier dysfunction through activated brain pericytes releasing inflammatory mediators in vitro. *Microvas. Res.* 124 61–66. 10.1016/j.mvr.2019.03.005 30885616

[B32] Dozio V. Sanchez J.-C. (2018). Profiling the proteomic inflammatory state of human astrocytes using DIA mass spectrometry. *J. Neuroinflamm.* 15:331. 10.1186/s12974-018-1371-6 30501627 PMC6267034

[B33] Elvira R. Tan E. K. Zhou Z. D. (2025). Three-dimensional midbrain organoids: A next-generation tool for Parkinson’s disease modelling and drug discovery. *Stem Cell Res. Therapy* 16:502. 10.1186/s13287-025-04660-4 40999543 PMC12465556

[B34] Fan Y. Kong H. Shi X. Sun X. Ding J. Wu J. (2008). Hypersensitivity of aquaporin 4-deficient mice to 1-methyl-4-phenyl-1,2,3,6-tetrahydropyrindine and astrocytic modulation. *Neurobiol. Aging* 29 1226–1236. 10.1016/j.neurobiolaging.2007.02.015 17353068

[B35] Fang Y. Dai S. Jin C. Si X. Gu L. Song Z. (2022). Aquaporin-4 polymorphisms are associated with cognitive performance in Parkinson’s disease. *Front. Aging Neurosci.* 13:740491. 10.3389/fnagi.2021.740491 35356146 PMC8959914

[B36] Fardell C. Zettergren A. Ran C. Carmine Belin A. Ekman A. Sydow O. (2018). S100B polymorphisms are associated with age of onset of Parkinson’s disease. *BMC Med. Genet.* 19:42. 10.1186/s12881-018-0547-3 29529989 PMC5848451

[B37] Farkas E. De Jong G. I. de Vos R. A. I. Jansen Steur E. N. H. Luiten P. G. M. (2000). Pathological features of cerebral cortical capillaries are doubled in Alzheimer’s disease and Parkinson’s disease. *Acta Neuropathol.* 100 395–402. 10.1007/s004010000195 10985698

[B38] Faucheux B. A. Agid Y. Hirsch E. C. Bonnet A.-M. (1999). Blood vessels change in the mesencephalon of patients with Parkinson’s disease. *Lancet* 353 981–982. 10.1016/S0140-6736(99)00641-8 10459912

[B39] Fujita K. Peng S. Ma Y. Tang C. C. Hellman M. Feigin A. (2021). Blood-brain barrier permeability in Parkinson’s disease patients with and without dyskinesia. *J. Neurol.* 268, 2246–2255. 10.1007/s00415-021-10411-1 33502551 PMC11197155

[B40] Gray M. T. Woulfe J. M. (2015). Striatal blood–brain barrier permeability in Parkinson’s disease. *J. Cereb. Blood Flow Metab.* 35 747–750. 10.1038/jcbfm.2015.32 25757748 PMC4420870

[B41] Guan J. Pavlovic D. Dalkie N. Waldvogel H. J. O’Carroll S. J. Green C. R. (2013). Vascular degeneration in Parkinson’s disease. *Brain Pathol.* 23 154–164. 10.1111/j.1750-3639.2012.00628.x 22897695 PMC8029297

[B42] Harms A. S. Ferreira S. A. Romero-Ramos M. (2021). Periphery and brain, innate and adaptive immunity in Parkinson’s disease. *Acta Neuropathol.* 141 527–545. 10.1007/s00401-021-02268-5 33555429 PMC7952334

[B43] Haussermann P. Kuhn W. Przuntek H. Muller T. (2001). Integrity of the blood-cerebrospinal fluid barrier in early Parkinson’s disease. *Neurosci. Lett.* 300, 182–184. 10.1016/S0304-3940(01)01574-9 11226641

[B44] Hélie-Legoupil P. Kloster F. Pareja J. Vladymyrov M. Mapunda J. A. Bouillet E. (2025). *In vivo* imaging of the barrier properties of the glia limitans during health and neuroinflammation. *Nat. Commun.* 16:8895. 10.1038/s41467-025-63945-7 41057305 PMC12504616

[B45] Hohoff C. Ponath G. Freitag C. M. Kästner F. Krakowitzky P. Domschke K. (2010). Risk variants in the S100B gene predict elevated S100B serum concentrations in healthy individuals. *Am. J. Med. Genet. Part B: Neuropsychiatric Genet.* 153B 291–297. 10.1002/ajmg.b.30950 19330775

[B46] Hourfar H. Aliakbari F. Aqdam S. R. Nayeri Z. Bardania H. Otzen D. E. (2023). The impact of α-synuclein aggregates on blood-brain barrier integrity in the presence of neurovascular unit cells. *Intern. J. Biol. Macromol.* 229 305–320. 10.1016/j.ijbiomac.2022.12.134 36535359

[B47] Huang J. Ding J. Wang X. Gu C. He Y. Li Y. (2022). Transfer of neuron-derived α-synuclein to astrocytes induces neuroinflammation and blood–brain barrier damage after methamphetamine exposure: Involving the regulation of nuclear receptor-associated protein 1. *Brain Behav. Immun.* 106 247–261. 10.1016/j.bbi.2022.09.002 36089218

[B48] Hurley M. J. Brandon B. Gentleman S. M. Dexter D. T. (2013). Parkinson’s disease is associated with altered expression of CaV1 channels and calcium-binding proteins. *Brain* 136 2077–2097. 10.1093/brain/awt134 23771339

[B49] Issidorides M. R. (1971). Neuronal vascular relationships in the zona compacta of normal and parkinsonian substantia nigra. *Brain Res.* 25 289–299. 10.1016/0006-8993(71)90439-2 4927145

[B50] Jagadeesan S. Workman M. J. Herland A. Svendsen C. N. Vatine G. D. (2020). Generation of a human iPSC-Based blood-brain barrier chip. *J. Visual. Exp.* e60925. 10.3791/60925 32176199

[B51] Janelidze S. Lindqvist D. Francardo V. Hall S. Zetterberg H. Blennow K. (2015). Increased CSF biomarkers of angiogenesis in Parkinson disease. *Neurology* 85 1834–1842. 10.1212/WNL.0000000000002151 26511451 PMC4662706

[B52] Jeon M.-T. Cogill S. A. Kim K.-S. Kim Y. Kim H. Lee C.-Y. (2025). TNF-α-NF-κB activation through pathological α-Synuclein disrupts the BBB and exacerbates axonopathy. *Cell Rep.* 44:116001. 10.1016/j.celrep.2025.116001 40652513

[B53] Kim S. Pajarillo E. Nyarko-Danquah I. Aschner M. Lee E. (2023). Role of astrocytes in Parkinson’s disease associated with genetic mutations and neurotoxicants. *Cells* 12:622. 10.3390/cells12040622 36831289 PMC9953822

[B54] Knott C. Wilkin G. P. Stern G. (1999). Astrocytes and microglia in the substantia nigra and caudate-putamen in Parkinson’s disease. *Parkinson. Related Disord.* 5 115–122. 10.1016/S1353-8020(99)00022-X 18591130

[B55] Kortekaas R. Leenders K. L. van Oostrom J. C. H. Vaalburg W. Bart J. Willemsen A. T. M. (2005). Blood–brain barrier dysfunction in parkinsonian midbrain *in vivo*. *Ann. Neurol.* 57 176–179. 10.1002/ana.20369 15668963

[B56] Kuan W.-L. Bennett N. He X. Skepper J. N. Martynyuk N. Wijeyekoon R. (2016). α-Synuclein pre-formed fibrils impair tight junction protein expression without affecting cerebral endothelial cell function. *Exp. Neurol.* 285 72–81. 10.1016/j.expneurol.2016.09.003 27632900

[B57] Lakhan S. E. Kirchgessner A. Tepper D. Leonard A. (2013). Matrix metalloproteinases and blood-brain barrier disruption in acute ischemic stroke. *Front. Neurol.* 4:32. 10.3389/fneur.2013.00032 23565108 PMC3615191

[B58] Lan G. Wang P. Chan R. B. Liu Z. Yu Z. Liu X. (2021). Astrocytic VEGFA: An essential mediator in blood–brain-barrier disruption in Parkinson’s disease. *Glia* 70 337–353. 10.1002/glia.24109 34713920

[B59] Lapshina K. V. Ekimova I. V. (2024). Aquaporin-4 and Parkinson’s disease. *Intern. J. Mol. Sci.* 25:1672. 10.3390/ijms25031672 38338949 PMC10855351

[B60] Lau A. So R. W. L. Lau H. H. C. Sang J. C. Ruiz-Riquelme A. Fleck S. C. (2020). α-Synuclein strains target distinct brain regions and cell types. *Nat. Neurosci.* 23 21–31. 10.1038/s41593-019-0541-x 31792467 PMC6930851

[B61] Lau K. Kotzur R. Richter F. (2024). Blood–brain barrier alterations and their impact on Parkinson’s disease pathogenesis and therapy. *Trans. Neurodegenerat.* 13:37. 10.1186/s40035-024-00430-z 39075566 PMC11285262

[B62] Lau K. Porschen L. T. Richter F. Gericke B. (2023). Microvascular blood-brain barrier alterations in isolated brain capillaries of mice over-expressing alpha-synuclein (Thy1-aSyn line 61). *Neurobiol. Dis.* 187:106298. 10.1016/j.nbd.2023.106298 37716515

[B63] Lee J. H. Han J. Kim H. Park S. M. Joe E. Jou I. (2019). Parkinson’s disease-associated LRRK2-G2019S mutant acts through regulation of SERCA activity to control ER stress in astrocytes. *Acta Neuropathol. Commun.* 7:68. 10.1186/s40478-019-0716-4 31046837 PMC6498585

[B64] Lin J. Ou R. Li C. Hou Y. Zhang L. Wei Q. (2023). Plasma glial fibrillary acidic protein as a biomarker of disease progression in Parkinson’s disease: A prospective cohort study. *BMC Med.* 21:420. 10.1186/s12916-023-03120-1 37932720 PMC10626747

[B65] Lindström V. Gustafsson G. Sanders L. H. Howlett E. H. Sigvardson J. Kasrayan A. (2017). Extensive uptake of α-synuclein oligomers in astrocytes results in sustained intracellular deposits and mitochondrial damage. *Mol. Cell. Neurosci.* 82 143–156. 10.1016/j.mcn.2017.04.009 28450268

[B66] Liu Y. Jiang H. Qin X. Tian M. Zhang H. (2022). PET imaging of reactive astrocytes in neurological disorders. *Eur. J. Nuclear Med. Mol. Imag.* 49 1275–1287. 10.1007/s00259-021-05640-5 34873637 PMC8921128

[B67] Llorens F. Schmitz M. Gloeckner S. F. Kaerst L. Hermann P. Schmidt C. (2015). Increased albumin CSF/serum ratio in dementia with Lewy bodies. *J. Neurol. Sci.* 358 398–403. 10.1016/j.jns.2015.10.011 26476775

[B68] Manu D. R. Slevin M. Barcutean L. Forro T. Boghitoiu T. Balasa R. (2023). Astrocyte involvement in blood-brain barrier function: a critical update highlighting novel, complex, neurovascular interactions. *Int. J. Mol. Sci.* 24:17146. 10.3390/ijms242417146 38138976 PMC10743219

[B69] Michinaga S. Koyama Y. (2019). Dual roles of astrocyte-derived factors in regulation of blood-brain barrier function after brain damage. *Intern. J. Mol. Sci.* 20:571. 10.3390/ijms20030571 30699952 PMC6387062

[B70] Mishra J. Jhun B. S. Hurst S. O-Uchi J. Csordás G. Sheu S.-S. (2017). The mitochondrial Ca^2 +^ uniporter: structure, function, and pharmacology. *Handb. Exp. Pharmacol.* 240, 129–156. 10.1007/164_2017_1 28194521 PMC5554456

[B71] Nanclares C. Poynter J. Martell-Martinez H. A. Vermilyea S. Araque A. Kofuji P. (2023). Dysregulation of astrocytic Ca2+ signaling and gliotransmitter release in mouse models of α-synucleinopathies. *Acta Neuropathol.* 145 597–610. 10.1007/s00401-023-02547-3 36764943 PMC10119048

[B72] Orr C. F. Rowe D. B. Mizuno Y. Mori H. Halliday G. M. (2005). A possible role for humoral immunity in the pathogenesis of Parkinson’s disease. *Brain* 128 2665–2674. 10.1093/brain/awh625 16219675

[B73] Papouin T. Dunphy J. Tolman M. Foley J. C. Haydon P. G. (2017). Astrocytic control of synaptic function. *Philos. Trans. R. Soc. Lond. B. Biol. Sci.* 372:20160154. 10.1098/rstb.2016.0154 28093548 PMC5247586

[B74] Papuć E. Rejdak K. (2020). Increased cerebrospinal fluid s100b and nse reflect neuronal and glial damage in Parkinson’s disease. *Front. Aging Neurosci.* 12:156. 10.3389/fnagi.2020.00156 32792937 PMC7387568

[B75] Parker C. A. Nabulsi N. Holden D. Lin S. Cass T. Labaree D. (2014). Evaluation of ^11^ C-BU99008, a PET ligand for the imidazoline_2_ binding sites in rhesus brain. *J. Nuclear Med.* 55 838–844. 10.2967/jnumed.113.131854 24711648

[B76] Paul G. Elabi O. F. (2022). Microvascular changes in Parkinson’s disease- focus on the neurovascular unit. *Front. Aging Neurosci.* 14:853372. 10.3389/fnagi.2022.853372 35360216 PMC8960855

[B77] Pediaditakis I. Kodella K. R. Manatakis D. V. Le C. Y. Hinojosa C. D. Tien-Street W. (2021). Modeling alpha-synuclein pathology in a human brain-chip to assess blood-brain barrier disruption. *Nat. Commun.* 12:5907. 10.1038/s41467-021-26066-5 34625559 PMC8501050

[B78] Pienaar I. S. Lee C. H. Elson J. L. McGuinness L. Gentleman S. M. Kalaria R. N. (2015). Deep-brain stimulation associates with improved microvascular integrity in the subthalamic nucleus in Parkinson’s disease. *Neurobiol. Dis.* 74 392–405. 10.1016/j.nbd.2014.12.006 25533682

[B79] Pisani V. Stefani A. Pierantozzi M. Natoli S. Stanzione P. Franciotta D. (2012). Increased blood-cerebrospinal fluid transfer of albumin in advanced Parkinson’s disease. *J. Neuroinflamm.* 9:670. 10.1186/1742-2094-9-188 22870899 PMC3441323

[B80] Pivoriûnas A. Verkhratsky A. (2021). Astrocyte–Endotheliocyte axis in the regulation of the blood–brain barrier. *Neurochem. Res.* 46 2538–2550. 10.1007/s11064-021-03338-6 33961207

[B81] Rite I. Machado A. Cano J. Venero J. L. (2007). Blood–brain barrier disruption induces *in vivo* degeneration of nigral dopaminergic neurons. *J. Neurochem.* 101 1567–1582. 10.1111/j.1471-4159.2007.04567.x 17437543

[B82] Sathe K. Maetzler W. Lang J. D. Mounsey R. B. Fleckenstein C. Martin H. L. (2012). S100B is increased in Parkinson’s disease and ablation protects against MPTP-induced toxicity through the RAGE and TNF-α pathway. *Brain: A J. Neurol.* 135(Pt 11), 3336–3347. 10.1093/brain/aws250 23169921 PMC3501971

[B83] Sha L. Zhao Y. Li S. Wei D. Tao Y. Wang Y. (2024). Insights to Ang/Tie signaling pathway: Another rosy dawn for treating retinal and choroidal vascular diseases. *J. Trans. Med.* 22:898. 10.1186/s12967-024-05441-y 39367441 PMC11451039

[B84] Siciliano B. Henkel N. D. Ryan V. W. G. Imami A. S. Vergis J. M. Xu C. (2025). Proinflammatory transcriptomic and kinomic alterations in astrocytes derived from patients with familial Alzheimer’s disease. *Brain Behav. Immun. Health* 47:101044. 10.1016/j.bbih.2025.101044 40656638 PMC12246863

[B85] Sofroniew M. V. Vinters H. V. (2010). Astrocytes: Biology and pathology. *Acta Neuropathol.* 119 7–35. 10.1007/s00401-009-0619-8 20012068 PMC2799634

[B86] Sonninen T.-M. Hämäläinen R. H. Koskuvi M. Oksanen M. Shakirzyanova A. Wojciechowski S. (2020). Metabolic alterations in Parkinson’s disease astrocytes. *Sci. Rep.* 10:14474. 10.1038/s41598-020-71329-8 32879386 PMC7468111

[B87] Sorrentino Z. A. Brooks M. M. T. Hudson V. Rutherford N. J. Golde T. E. Giasson B. I. (2017). Intrastriatal injection of α-synuclein can lead to widespread synucleinopathy independent of neuroanatomic connectivity. *Mol. Neurodegenerat.* 12:40. 10.1186/s13024-017-0182-z 28552073 PMC5447308

[B88] Sorrentino Z. A. Giasson B. I. Chakrabarty P. (2020). α-Synuclein and astrocytes: Tracing the pathways from homeostasis to neurodegeneration in Lewy body disease. *Acta Neuropathol.* 138 1–21. 10.1007/s00401-019-01977-2 30798354 PMC6571045

[B89] Spampinato S. F. Bortolotto V. Canonico P. L. Sortino M. A. Grilli M. (2019). Astrocyte-Derived paracrine signals: Relevance for neurogenic niche regulation and blood–brain barrier integrity. *Front. Pharmacol.* 10:1346. 10.3389/fphar.2019.01346 31824311 PMC6881379

[B90] Stern M. (1991). The epidemiology of Parkinson’s disease. *Arch. Neurol.* 48:903. 10.1001/archneur.1991.00530210029018 1953412

[B91] Stevenson T. J. Johnson R. H. Savistchenko J. Rustenhoven J. Woolf Z. Smyth L. C. D. (2022). Pericytes take up and degrade α-synuclein but succumb to apoptosis under cellular stress. *Sci. Rep.* 12:17314. 10.1038/s41598-022-20261-0 36243723 PMC9569325

[B92] Su W. Chen H. B. Li S. H. Wu D. Y. (2012). Correlational study of the serum levels of the glial fibrillary acidic protein and neurofilament proteins in Parkinson’s disease patients. *Clin. Neurol. Neurosurg.* 114 372–375. 10.1016/j.clineuro.2011.11.002 22206859

[B93] Subburaman T. T. (2010). Neuroprotective action of piper longum against MPTP-induced changes in mouse brain. *Ann. Neurosci.* 17 18–21. 10.5214/ans.0972-7531.2010.170102

[B94] Sui Y.-T. Bullock K. M. Erickson M. A. Zhang J. Banks W. A. (2014). Alpha synuclein is transported into and out of the brain by the blood–brain barrier. *Peptides* 62 197–202. 10.1016/j.peptides.2014.09.018 25278492 PMC4378645

[B95] Sun H. Liang R. Yang B. Zhou Y. Liu M. Fang F. (2016). Aquaporin-4 mediates communication between astrocyte and microglia: Implications of neuroinflammation in experimental Parkinson’s disease. *Neuroscience* 317 65–75. 10.1016/j.neuroscience.2016.01.003 26774050

[B96] Tang Y. Han L. Li S. Hu T. Xu Z. Fan Y. (2023). Plasma GFAP in Parkinson’s disease with cognitive impairment and its potential to predict conversion to dementia. *NPJ Parkinson’s Dis.* 9:23. 10.1038/s41531-023-00447-7 36759508 PMC9911758

[B97] Thamizh Thenral S. Vanisree A. J. (2012). Peripheral assessment of the genes AQP4, PBP and TH in patients with Parkinson’s disease. *Neurochem. Res.* 37 512–515. 10.1007/s11064-011-0637-5 22083667

[B98] Tomás-Camardiel M. Rite I. Herrera A. J. de Pablos R. M. Cano J. Machado A. (2004). Minocycline reduces the lipopolysaccharide-induced inflammatory reaction, peroxynitrite-mediated nitration of proteins, disruption of the blood–brain barrier, and damage in the nigral dopaminergic system. *Neurobiol. Dis.* 16 190–201. 10.1016/j.nbd.2004.01.010 15207276

[B99] Tong J. Ang L.-C. Williams B. Furukawa Y. Fitzmaurice P. Guttman M. (2015). Low levels of astroglial markers in Parkinson’s disease: Relationship to α-synuclein accumulation. *Neurobiol. Dis.* 82 243–253. 10.1016/j.nbd.2015.06.010 26102022 PMC4641013

[B100] Tsai C. H. Lo S. K. See L. C. Chen H. Z. Chen R. S. Weng Y. H. (2002). Environmental risk factors of young onset Parkinson’s disease: A case-control study. *Clin. Neurol. Neurosurg.* 104 328–333. 10.1016/S0303-8467(02)00027-6 12140099

[B101] Tyacke R. J. Myers J. F. M. Venkataraman A. Mick I. Turton S. Passchier J. (2018). Evaluation of ^11^ C-BU99008, a PET Ligand for the Imidazoline _2_ Binding Site in Human Brain. *J. Nuclear Med.* 59 1597–1602. 10.2967/jnumed.118.208009 29523627

[B102] Valles S. L. Singh S. K. Campos-Campos J. Colmena C. Campo-Palacio I. Alvarez-Gamez K. (2023). Functions of astrocytes under normal conditions and after a brain disease. *Int. J. Mol. Sci.* 24:8434. 10.3390/ijms24098434 37176144 PMC10179527

[B103] Van der Perren A. Gelders G. Fenyi A. Bousset L. Brito F. Peelaerts W. (2020). The structural differences between patient-derived α-synuclein strains dictate characteristics of Parkinson’s disease, multiple system atrophy and dementia with Lewy bodies. *Acta Neuropathol.* 139 977–1000. 10.1007/s00401-020-02157-3 32356200 PMC7244622

[B104] Vizuete M. L. Venero J. L. Vargas C. Ilundáin A. A. Echevarra M. Machado A. (1999). Differential upregulation of Aquaporin-4 mRNA expression in reactive astrocytes after brain injury: Potential role in brain edema. *Neurobiol. Dis.* 6 245–258. 10.1006/nbdi.1999.0246 10448052

[B105] Wachter B. Schürger S. Schmid A. Gröger A. Sadler R. Speidel A. (2012). 6-Hydroxydopamine leads to T2 hyperintensity, decreased claudin-3 immunoreactivity and altered aquaporin 4 expression in the striatum. *Behav. Brain Res.* 232 148–158. 10.1016/j.bbr.2012.04.005 22516842

[B106] Wada K. Arai H. Takanashi M. Fukae J. Oizumi H. Yasuda T. (2006). Expression levels of vascular endothelial growth factor and its receptors in Parkinson’s disease. *NeuroReport* 17 705–709. 10.1097/01.wnr.0000215769.71657.65 16641673

[B107] Wang C. Yang T. Liang M. Xie J. Song N. (2021). Astrocyte dysfunction in Parkinson’s disease: From the perspectives of transmitted α-synuclein and genetic modulation. *Trans. Neurodegenerat.* 10:39. 10.1186/s40035-021-00265-y 34657636 PMC8522040

[B108] Wang P. Ye Y. (2021). Astrocytes in neurodegenerative diseases: A perspective from tauopathy and α-Synucleinopathy. *Life* 11:938. 10.3390/life11090938 34575087 PMC8471224

[B109] Wang T. Sun Y. Dettmer U. (2023). Astrocytes in Parkinson’s disease: From role to possible intervention. *Cells* 12:2336. 10.3390/cells12192336 37830550 PMC10572093

[B110] Wang X. Li W. Zhao X. Hu N. Wang X. Xiao X. (2024). Dysregulated coagulation in Parkinson’s disease. *Cells* 13:1874. 10.3390/cells13221874 39594622 PMC11592531

[B111] Williams G. P. Schonhoff A. M. Jurkuvenaite A. Gallups N. J. Standaert D. G. Harms A. S. (2021). CD4 T cells mediate brain inflammation and neurodegeneration in a mouse model of Parkinson’s disease. *Brain* 144 2047–2059. 10.1093/brain/awab103 33704423 PMC8370411

[B112] Wilson H. Dervenoulas G. Pagano G. Tyacke R. J. Polychronis S. Myers J. (2019). Imidazoline 2 binding sites reflecting astroglia pathology in Parkinson’s disease: An *in vivo*11C-BU99008 PET study. *Brain* 142 3116–3128. 10.1093/brain/awz260 31504212

[B113] Woodard C. M. Campos B. A. Kuo S.-H. Nirenberg M. J. Nestor M. W. Zimmer M. (2014). iPSC-Derived dopamine neurons reveal differences between monozygotic twins discordant for Parkinson’s disease. *Cell Rep.* 9 1173–1182. 10.1016/j.celrep.2014.10.023 25456120 PMC4255586

[B114] Xue X. Zhang W. Zhu J. Chen X. Zhou S. Xu Z. (2019). Aquaporin-4 deficiency reduces TGF−β1 in mouse midbrains and exacerbates pathology in experimental Parkinson’s disease. *J. Cell. Mol. Med.* 23 2568–2582. 10.1111/jcmm.14147 30680924 PMC6433854

[B115] Yamashita K. Y. Bhoopatiraju S. Silverglate B. D. Grossberg G. T. (2023). Biomarkers in Parkinson’s disease: A state of the art review. *Biomarkers Neuropsychiatry* 9:100074. 10.1016/j.bionps.2023.100074

[B116] Yang P. Pavlovic D. Waldvogel H. Dragunow M. Synek B. Turner C. (2015). String vessel formation is increased in the brain of Parkinson disease. *J. Parkinson’s Dis.* 5 821–836. 10.3233/JPD-140454 26444086

[B117] Zarate S. M. Huntington T. E. Bagher P. Srinivasan R. (2023). Aging reduces calreticulin expression and alters spontaneous calcium signals in astrocytic endfeet of the mouse dorsolateral striatum. *NPJ Aging* 9:5. 10.1038/s41514-023-00102-8 37002232 PMC10066375

[B118] Zhang Y. Zhang C. He X.-Z. Li Z.-H. Meng J.-C. Mao R.-T. (2023). Interaction between the glymphatic system and α-Synuclein in Parkinson’s disease. *Mol. Neurobiol.* 60 2209–2222. 10.1007/s12035-023-03212-2 36637746

